# Association between preoperative physical exercise and the risk of postoperative venous thromboembolism: nationwide population-based cohort study

**DOI:** 10.1093/bjsopen/zrag109

**Published:** 2026-09-10

**Authors:** Chan-Sik Kim, Bumwoo Park, Hyeong-Seok Yoon, Ji-Hoon Sim

**Affiliations:** Department of Anesthesiology and Pain Medicine, Asan Medical Center, University of Ulsan College of Medicine, Seoul, Republic of Korea; Department of Information Medicine, Asan Medical Center, University of Ulsan College of Medicine, Seoul, Republic of Korea; Department of Anesthesiology and Pain Medicine, Gangneung Asan Hospital, University of Ulsan College of Medicine, Gangneung, Republic of Korea; Department of Anesthesiology and Pain Medicine, Asan Medical Center, University of Ulsan College of Medicine, Seoul, Republic of Korea

**Keywords:** venous thromboembolism, deep vein thrombosis, exercise, prehabilitation, population-based cohort

## Abstract

**Background:**

Although preoperative exercise is widely promoted within prehabilitation programmes, the association between habitual preoperative exercise and postoperative venous thromboembolism (VTE) remains unclear. This study examined the association between the frequency of preoperative exercise and postoperative VTE risk.

**Methods:**

Using the National Health Insurance Service–National Sample Cohort (2009–2016), adults who underwent surgery and preoperative health screening were identified. Exercise was classified as exerciser *versus* non-exerciser and by frequency (none, 1–2, or ≥3 times/week). Multivariable logistic regression adjusted for demographic, clinical, and surgical factors was used to estimate VTE risk, with subgroup analyses across demographic and lifestyle strata.

**Results:**

A total of 118 340 patients were enrolled in this study and categorized based on their exercise habits: non-exercisers (*n* = 33 351; 28.2%) and exercisers (*n* = 84 989; 71.8%). Engagement in preoperative exercise was significantly associated with a reduced risk of postoperative VTE (adjusted odds ratio [aOR]: 0.69, 95% confidence interval [c.i.]: 0.49–0.99, *P* = 0.041) compared to the non-exerciser group. A clear dose-response relationship was evident; while exercising 1–2 times per week was associated with a lower but non-significant risk (aOR: 0.79, 95% c.i.: 0.48–1.26, *P* = 0.334), the lowest and statistically significant risk was observed among patients exercising ≥3 times per week (aOR: 0.66, 95% c.i.: 0.45–0.96, *P* = 0.031, *P* for trend = 0.032). Secondary analyses confirmed similar protective trends for deep vein thrombosis, with varying degrees of risk reduction observed for other complications such as stroke. Subgroup analysis indicated that the impact of exercise on outcomes was more pronounced in male and smoking patients.

**Conclusions:**

Preoperative exercise is an independent, modifiable factor associated with a dose-dependent reduction in VTE risk, highlighting its role as a non-pharmacological strategy to enhance surgical safety.

## Teaser

In a nationwide cohort of 118 340 surgical patients from the Korean National Health Insurance Service–National Sample Cohort, habitual preoperative exercise was independently associated with a 31% lower risk of postoperative venous thromboembolism (VTE), with a clear frequency-dependent gradient. The strongest protection was observed among patients exercising three or more times per week, supporting the incorporation of preoperative exercise status into routine perioperative risk assessment as a modifiable, non-pharmacological factor.

## Introduction

Postoperative venous thromboembolism (VTE), which includes both deep vein thrombosis (DVT) and pulmonary embolism (PE), remains a leading cause of preventable morbidity and mortality in patients undergoing major surgery^1–4^. Despite widespread implementation of pharmacological and mechanical prophylaxis, VTE continues to pose a significant challenge, often leading to prolonged hospital stays, substantial healthcare costs, and an increased risk of sudden, catastrophic death^5,6^. In the perioperative setting, this risk is further exacerbated by a complex interplay of intraoperative immobilization, systemic inflammatory responses, and the prothrombotic state inherently induced by surgical trauma^7^. Consequently, identifying modifiable preoperative factors that can proactively reduce this risk is of paramount clinical and public health importance.

Emerging evidence has highlighted the concept of ‘prehabilitation’—the practice of optimizing a patient’s physiological reserve and functional capacity through targeted preoperative interventions to enhance postoperative recovery^8–10^. Among these strategies, physical exercise has been identified as a cornerstone, capable of augmenting cardiovascular fitness, attenuating systemic inflammation, and improving venous haemodynamics by strengthening the skeletal muscle pump^11–13^. While several high-impact studies have demonstrated the efficacy of preoperative exercise in reducing postoperative complications and shortening hospital stays—particularly after major abdominal or oncological surgeries—its specific protective role against VTE remains comparatively less explored^8,14–16^. Addressing this association is essential for establishing standardized, evidence-based preoperative care strategies, particularly for an increasingly aging and comorbid surgical population.

Therefore, utilizing a nationwide population-based cohort, the aim of this study was to evaluate the independent association between habitual preoperative exercise frequency—ascertained as a self-reported baseline characteristic from routine national health screening, rather than a prescribed or supervised prehabilitation intervention—and the risk of postoperative VTE. Furthermore, the potential dose-response relationship between exercise frequency and VTE prevention was investigated to provide refined recommendations for preoperative risk management and patient optimization.

## Material and methods

### Data source and study population

Data for this population-based cohort study were obtained from the South Korean National Health Insurance Service–National Sample Cohort (NHIS-NSC). This database comprises a representative 2.2% sample of the national population, constructed by the National Health Insurance Service using stratified random sampling based on age, sex, region, and income; this stratification is an inherent design feature of the database and was not performed by the investigators. The NHIS-NSC provides comprehensive longitudinal records, including demographic profiles, inpatient and outpatient healthcare services, surgical interventions, ICD-10-coded clinical diagnoses, and pharmacological prescriptions. Additionally, all-cause mortality data are integrated via linkage with Statistics Korea^17^. To protect participant privacy, all datasets were fully de-identified prior to being made available for research. This study was conducted in accordance with the ethical principles of the Declaration of Helsinki. The Institutional Review Board of Asan Medical Center granted ethical approval (IRB No. 2025-0789) and authorized a waiver of informed consent considering the retrospective nature of the study using anonymized secondary data.

**Figure 1:**
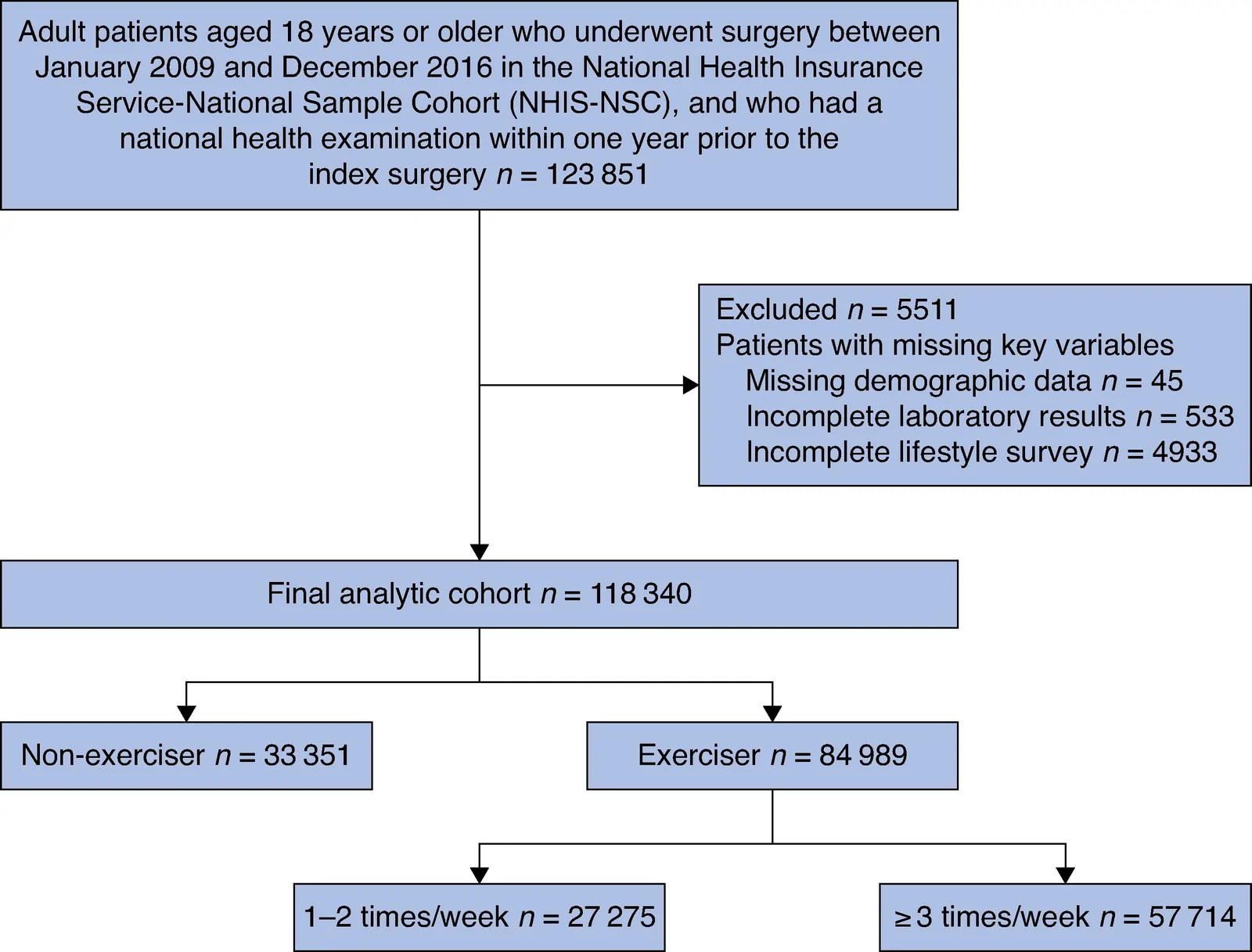
Flow diagram of the study population

Utilizing the National Health Insurance Service–National Sample Cohort (NHIS-NSC), a population of adults (aged ≥18 years) who underwent operative treatment between January 2009 and December 2016 were identified. To ensure the availability of baseline clinical profiles, inclusion was limited to individuals with a documented national health screening within the 12 months preceding the primary surgery. Because eligibility required attendance at routine health screening before a subsequently scheduled operation, the cohort predominantly comprises patients undergoing planned, elective surgery. From this group, candidates were excluded if they lacked essential data, specifically regarding demographics, laboratory results, or lifestyle factors such as tobacco use, alcohol consumption, and physical activity levels. The comprehensive screening and selection process is illustrated in the flowchart (*Fig. 1*).

### Data collection and variables

Baseline demographic and anthropometric data, including age, sex, body mass index (BMI), and waist circumference, were retrieved from national health screening records. Haemodynamic profiles, specifically systolic and diastolic blood pressure, were also obtained during these clinical examinations. Detailed biochemical profiles extracted from the health screening database included haemoglobin levels, fasting glucose, and total cholesterol, as well as liver function parameters such as aspartate aminotransferase (AST), alanine aminotransferase (ALT), and gamma-glutamyl transferase (GGT). Lifestyle-related information was collected from self-reported health surveys, which provided data on smoking status (non-smoker, ex-smoker, or current smoker), drinking frequency (none, <1 time/week, 1–2 times/week, or ≥3 times/week), and physical activity levels categorized by exercise frequency (none, 1–2 times/week, or ≥3 times/week).

Socioeconomic status was determined based on insurance type, categorized as regional insured, workplace insured, or medical aid beneficiary, and income level, divided into quartiles (Q1–Q4). Institutional data included hospital size, defined by bed capacity (<100, 100–499, ≥500 beds), and the year of surgery, recorded in biennial periods from 2009 to 2016. Surgical data were classified by specialty, including general, otolaryngological, urologic, orthopaedic, gynaecologic, neurosurgical, and cardiothoracic surgeries, with general, orthopaedic, neurosurgical, and cardiothoracic procedures designated as high-risk surgery, defined as major surgical categories considered to carry a higher risk of postoperative complications, including VTE. The presence of preexisting medical conditions was identified using ICD-10 codes recorded during the preoperative period, covering myocardial infarction, congestive heart failure, peripheral vascular disease, cerebrovascular disease, dementia, chronic pulmonary disease, rheumatic disease, peptic ulcer disease, liver disease, diabetes, hemiplegia/paraplegia, renal disease, and various malignancies, including metastatic solid tumours, as detailed in *Table S1*. These comorbidities were further used to calculate the Charlson Comorbidity Index (CCI) score, categorized as 0–2, 3–4, or ≥5.

### Exercise exposure and clinical outcomes

The primary exposure of interest was the frequency of preoperative exercise, obtained from national health screening records documented within the year preceding the index surgery. Accordingly, this exposure represents a patient’s habitual, self-reported exercise behaviour at baseline and does not reflect a structured, prescribed, or supervised preoperative exercise (prehabilitation) intervention. Participants were categorized into two groups: non-exercisers and exercisers. Participants were further stratified by exercise frequency into three levels: none, 1–2 times per week, and ≥3 times per week, to investigate potential dose-response relationships across all evaluated endpoints. This three-level grouping reflects categories commonly applied in studies using the Korean national health screening data. To allow a more granular evaluation of the dose–response relationship, exercise frequency was additionally analysed as a four-level variable (none, 1–2, 3–4, and ≥5 times per week). Exercise frequency was self-reported at the national health screening examination and refers to the respondent’s usual weekly frequency of regular exercise.

The primary outcome was the association between these exercise patterns and the development of postoperative venous thromboembolism (VTE), defined as the pre-specified composite of deep vein thrombosis (DVT) and pulmonary embolism (PE). Because DVT accounted for the large majority of VTE events (123 of 133; 92.5%), DVT was additionally examined as a separate secondary outcome; PE occurred in only 14 patients and, owing to this small number, was not analysed as a separate outcome. Secondary outcomes included the relationship between exercise status and various postoperative complications (for example deep vein thrombosis, stroke, pneumonia, and surgical site infection), healthcare utilization (30-day hospital readmission rates), all-cause mortality at one year, and overall mortality throughout the entire follow-up period. Subgroup analyses were conducted to determine whether the associations between exercise and these postoperative outcomes differed based on key patient characteristics such as age, sex, and lifestyle factors. All clinical endpoints were identified using standardized claims-based algorithms and diagnosis codes from the NHIS-NSC database (*Table S2*). Consistency was ensured through predefined criteria and designated postoperative observation windows to capture new diagnoses.

### Statistical analysis

Baseline characteristics were compared according to exercise frequency, analyzed both as a binary variable (non-exerciser *versus* exerciser) and across three categories (none, 1–2 times/week, and ≥3 times/week). Continuous variables are reported as means with standard deviations (SD) or medians with interquartile ranges (IQR), depending on their distribution, while categorical variables are presented as frequencies and percentages. Differences between groups were assessed using one-way analysis of variance or the Kruskal–Wallis test for continuous variables, and the chi-square test for categorical variables. To evaluate the balance of covariates between groups, standardized mean differences (SMDs) were calculated, with the maximum absolute difference reported.

To estimate the relationship between preoperative exercise and postoperative outcomes, multivariable logistic regression was employed for complications (VTE, DVT, stroke, pneumonia, and surgical site infection) and Cox proportional hazards models for mortality and long-term follow-up data. Odds ratios (ORs) and hazard ratios (HRs) with 95% confidence intervals (CIs) were calculated, using the non-exerciser (or ‘none’ frequency) group as the reference. Three hierarchical models were constructed to evaluate the independence of this association. Covariates were entered in a pre-specified, hierarchical sequence—demographic factors first, followed by clinical and laboratory measures, and finally comorbidities and lifestyle factors—so that the stability of the exercise–VTE association could be assessed across progressively more complete adjustment. Model 1 adjusted for age and sex. Model 2 included the variables from Model 1 plus BMI, haemoglobin levels, systolic blood pressure, and the CCI. Model 3 further incorporated lifestyle factors (smoking status and alcohol consumption), surgical risk (high-risk surgery), and specific medical histories, including congestive heart failure, cerebrovascular disease, chronic pulmonary disease, diabetes, and renal disease. Based on the fully adjusted estimates from Model 3, forest plots were generated to illustrate the effect sizes and dose-response trends for the three exercise categories across all studied outcomes.

To investigate potential dose-response relationships, a *P* for trend analysis was performed by treating the three exercise frequency categories as a continuous variable in the models. Additionally, subgroup analyses were performed to assess the consistency of the association between binary exercise status (non-exerciser *versus* exerciser) and postoperative outcomes across strata of age (<65 *versus* ≥65 years), sex (female *versus* male), smoking status (non-smoker *versus* smoker), and drinking frequency (non-drinker *versus* drinker).

All statistical analyses were conducted using R software (version 4.4.2) and SAS software (version 9.4; SAS Institute, Cary, NC, USA). Statistical significance was assessed using two-sided tests with a significance threshold of *P* < 0.05.

## Results

### Baseline characteristics according to exercise status

A total of 118 340 patients were enrolled in this study and categorized based on their exercise habits: non-exercisers (*n* = 33 351; 28.2%) and exercisers (*n* = 84 989; 71.8%) (*Fig. 1* and *Table 1*). Among exercisers, 27 275 patients (23.0% of the cohort) reported exercising 1–2 times per week and 57 714 (48.8%) reported exercising ≥3 times per week. The cohort had a mean age of 50.0 (14.0) years, with a male predominance of 55.7% (*n* = 65 872). Regarding institutional capacity, the vast majority of procedures (96.4%) were conducted at facilities with fewer than 100 beds, while only 1.5% and 2.1% took place in hospitals with 100–499 beds and ≥ 500 beds, respectively (*P* = 0.119). The study population was primarily covered by workplace-based insurance (77.6%), followed by regional insurance (22.4%) (*P* < 0.001). Socioeconomic status, measured by income quartiles, showed a distribution of 14.7% in Q1, 20.6% in Q2, 33.3% in Q3, and 31.4% in Q4 (*P* < 0.001).

**Table 1:** Baseline characteristics according to exercise status

	Total study population			
	Exercise status			
	Total (*n* = 118 340)	Non-exerciser (*n* = 33 351)	Exerciser (*n* = 84 989)	*P*
**Demographic variables**				
Age, years	50.0 ± 14.0	52.3 ± 13.7	49.1 ± 14.0	<0.001
Male sex	65 872 (55.7%)	17 416 (52.2%)	15 961 (58.5%)	<0.001
BMI, kg/m^2^	23.9 ± 3.3	23.9 ± 3.3	23.9 ± 3.2	0.042
Waist circumference, cm	80.9 ± 9.2	81.1 ± 9.2	80.8 ± 9.2	<0.001
Systolic blood pressure, mmHg	122.6 ± 14.8	123.2 ± 15.1	122.4 ± 14.7	<0.001
Diastolic blood pressure, mmHg	76.4 ± 10.0	76.6 ± 10.0	76.3 ± 9.9	<0.001
Haemoglobin, g/dl	14.0 ± 1.6	13.9 ± 1.6	14.1 ± 1.6	<0.001
Fasting glucose, mg/dl	99.1 ± 24.9	99.7 ± 25.4	98.9 ± 24.7	<0.001
Total cholesterol, mg/dl	194.9 ± 40.0	195.9 ± 40.1	194.5 ± 40.0	<0.001
AST, U/l	25.7 ± 25.5	26.0 ± 22.5	25.6 ± 26.6	0.007
ALT, U/l	25.6 ± 38.8	25.7 ± 28.2	25.6 ± 42.2	0.635
GGT, U/l	38.1 ± 52.4	38.8 ± 55.5	37.8 ± 51.1	0.002
Institutional volume				0.119
< 100 beds	114 128 (96.4%)	32 190 (96.5%)	81 938 (96.4%)	
100–499 beds	1786 (1.5%)	519 (1.6%)	1267 (1.5%)	
≥ 500 beds	2426 (2.1%)	642 (1.9%)	1784 (2.1%)	
Insurance type				<0.001
Regional insured	26 451 (22.4%)	8293 (24.9%)	18 158 (21.4%)	
Workplace insured	91 889 (77.6%)	25 058 (75.1%)	66 831 (78.6%)	
Income level				<0.001
Q1	17 391 (14.7%)	5383 (16.1%)	12 008 (14.1%)	
Q2	24 392 (20.6%)	7041 (21.1%)	17 351 (20.4%)	
Q3	39 420 (33.3%)	11 053 (33.1%)	28 367 (33.4%)	
Q4	37 137 (31.4%)	9874 (29.6%)	27 263 (32.1%)	
Year of surgery				<0.001
2009–2010	33 846 (28.6%)	10 583 (31.7%)	23 263 (27.4%)	
2011–2012	36 706 (31.0%)	10 668 (32.0%)	26 038 (30.6%)	
2013–2014	28 634 (24.2%)	7539 (22.6%)	21 095 (24.8%)	
2015–2016	19 154 (16.2%)	4561 (13.7%)	14 593 (17.2%)	
Smoking status				<0.001
Non-smoker	68 428 (57.8%)	20 515 (61.5%)	47 913 (56.4%)	
Ex-smoker	19 389 (16.4%)	4448 (13.3%)	14 941 (17.6%)	
Current smoker	30 523 (25.8%)	8388 (25.2%)	22 135 (26.0%)	
Frequency of drinking alcohol				<0.001
None	60 095 (50.8%)	19 648 (58.9%)	40 447 (47.6%)	
1–2 times/week	40 769 (34.5%)	8797 (26.4%)	31 972 (37.6%)	
3–4 times/week	12 491 (10.6%)	3196 (9.6%)	9295 (10.9%)	
≥5 times/week	4985 (4.2%)	1710 (5.1%)	3275 (3.9%)	
Type of surgery				<0.001
General	14 809 (12.5%)	4350 (13.0%)	10 459 (12.3%)	
Otolaryngology	24 553 (20.7%)	6349 (19.0%)	18 204 (21.4%)	
Urologic	10 320 (8.7%)	2840 (8.5%)	7480 (8.8%)	
Orthopaedic	42 739 (36.1%)	12 555 (37.6%)	30 184 (35.5%)	
Gynaecologic	11 343 (9.6%)	3295 (9.9%)	8048 (9.5%)	
Neurosurgery	4936 (4.2%)	1486 (4.5%)	3450 (4.1%)	
Cardiothoracic	1007 (0.9%)	284 (0.9%)	723 (0.9%)	
Other Surgeries	8633 (7.3%)	2192 (6.6%)	6441 (7.6%)	
High-risk surgery (general, orthopaedic, neurosurgery, and cardiothoracic surgery)	63 491 (53.7%)	18 675 (56.0%)	40 173 (47.3%)	<0.001
Myocardial infarction	737 (0.6%)	232 (0.7%)	505 (0.6%)	0.042
Congestive heart failure	2048 (1.7%)	729 (2.2%)	1319 (1.6%)	<0.001
Peripheral vascular disease	8384 (7.1%)	2824 (8.5%)	5560 (6.5%)	<0.001
Cerebrovascular disease	5183 (4.4%)	1800 (5.4%)	3383 (4.0%)	<0.001
Dementia	270 (0.2%)	90 (0.3%)	180 (0.2%)	<0.001
Chronic pulmonary disease	22 688 (19.2%)	6930 (20.8%)	15 758 (18.5%)	<0.001
Rheumatic disease	3051 (2.6%)	979 (2.9%)	2072 (2.4%)	<0.001
Peptic ulcer disease	24 037 (20.3%)	7566 (22.7%)	16 471 (19.4%)	<0.001
Mild liver disease	16 255 (13.7%)	4975 (14.9%)	11 280 (13.3%)	<0.001
Diabetes (no complication)	10 404 (8.8%)	3289 (9.9%)	7115 (8.4%)	<0.001
Diabetes (with complication)	4466 (3.8%)	1344 (4.0%)	3122 (3.7%)	<0.001
Hemiplegia or paraplegia	274 (0.2%)	103 (0.3%)	171 (0.2%)	<0.001
Renal disease	1523 (1.3%)	487 (1.5%)	1036 (1.2%)	<0.001
Any malignancy	4443 (3.8%)	1347 (4.0%)	3096 (3.6%)	<0.001
Moderate or severe liver disease	377 (0.3%)	113 (0.3%)	264 (0.3%)	0.473
Metastatic solid tumour	301 (0.3%)	79 (0.2%)	222 (0.3%)	0.494
AIDS or HIV	0 (0%)	0 (0%)	0 (0%)	1.000
CCI score				<0.001
0–2	103 499 (87.5%)	28 542 (85.6%)	74 957 (88.2%)	
3–4	11 281 (9.5%)	3613 (10.8%)	7668 (9.0%)	
≥5	3560 (3.0%)	1196 (3.6%)	2364 (2.8%)	
**Outcomes**				
VTE	133 (0.1%)	54 (0.2%)	79 (0.1%)	0.002
DVT	123 (0.1%)	51 (0.2%)	72 (0.1%)	0.001
Pulmonary embolism	14 (0.0%)	5 (0.0%)	2 (0.0%)	0.686
Stroke	1978 (1.7%)	720 (2.2%)	1258 (1.5%)	<0.001
Pneumonia	431 (0.4%)	133 (0.4%)	298 (0.4%)	0.237
Surgical site infection	986 (0.8%)	306 (0.9%)	680 (0.8%)	0.050
30-day readmission	827 (0.7%)	239 (0.7%)	588 (0.7%)	0.674
90-day mortality	56 (0.0%)	13 (0.0%)	43 (0.1%)	0.498
1-year mortality	288 (0.2%)	102 (0.3%)	186 (0.2%)	0.008
Overall mortality	5381 (4.5%)	2001 (6.0%)	3380 (4.0%)	<0.001

Values are expressed as mean ± standard deviation or *n* (proportion). BMI, body mass index; AST, Aspartate aminotransferase; ALT: Alanine aminotransferase; GGT: Gamma-glutamyl transferase; HIV, human immunodeficiency virus; AIDS, acquired immunodeficiency syndrome; CCI, Charlson comorbidity index; VTE, venous thromboembolism; DVT, deep vein thrombosis.

When comparing exercise status, non-exercisers were significantly older than exercisers (52.3 *versus* 49.1 years; *P* < 0.001). The proportion of male participants was higher in the exerciser group (58.5%) than in the non-exerciser group (52.2%) (*P* < 0.001). Clinical parameters, including systolic blood pressure (123.2 *versus* 122.4 mmHg) and fasting glucose levels (99.7 *versus* 98.9 mg/dL), were slightly but significantly higher in the non-exercising cohort (all *P* < 0.001).

Regarding comorbidities, non-exercisers exhibited a significantly higher prevalence of most pre-existing conditions compared to exercisers. Specifically, the rates of congestive heart failure (2.2% *versus* 1.6%), cerebrovascular disease (5.4% *versus* 4.0%), myocardial infarction (0.7% *versus* 0.6%), and chronic pulmonary disease (20.8% *versus* 18.5%) were all significantly higher in the non-exerciser group (all *P* < 0.05). This trend was also observed in diabetes mellitus, with non-exercisers showing significantly higher proportions of both uncomplicated (9.9% *versus* 8.4%) and complicated (4.0% *versus* 3.7%) cases (all *P* < 0.001). In contrast, no significant differences were found between the two groups for moderate to severe liver disease or metastatic solid tumours. Baseline characteristics of the study population, stratified by exercise frequency and by exerciser *versus* non-exerciser status, are detailed in *Table S3*.

### Primary outcome: VTE

The incidence of the primary outcome, VTE, was significantly higher in the non-exerciser group compared to the exerciser group (0.2% *versus* 0.1%; *P* = 0.002). Pulmonary embolism occurred in only 14 patients (0.01% of the cohort). *Table 2* and *Table S4* show the independent association between exercise and VTE risk across three adjustment models. Exercisers consistently demonstrated a lower risk of VTE than non-exercisers in Model 1 (OR: 0.68, 95% c.i.: 0.48–0.96; *P* = 0.027), Model 2 (OR: 0.69, 95% c.i.: 0.49–0.98; *P* = 0.037), and the fully adjusted Model 3 (OR: 0.69, 95% c.i.: 0.49–0.99; *P* = 0.041).

**Table 2:** Logistic regression analysis for the association between exercise and the risk of VTE

		Multivariable model 1	Multivariable mode 2	Multivariable model 3
Variable	Level	Adjusted OR (95% c.i.)	Adjusted OR (95% c.i.)	Adjusted OR (95% c.i.)
Exercise status	Non-exerciser	1.00 (reference)	1.00 (reference)	1.00 (reference)
	Exerciser	0.68 (0.48–0.96)	0.69 (0.49–0.98)	0.69 (0.49–0.99)
	*P*-value	0.027	0.037	0.041
Exercise frequency	None	1.00 (reference)	1.00 (reference)	1.00 (reference)
	1–2/week	0.77 (0.47–1.23)	0.78 (0.45–1.25)	0.79 (0.48–1.26)
	≥3/week	0.64 (0.44–0.94)	0.65 (0.45–0.96)	0.66 (0.45–0.96)
	*P* for trend	0.021	0.029	0.032

Model 1: Adjusted for age and sex. Model 2: Adjusted for Model 1 variables plus BMI, hemoglobin, systolic blood pressure, and CCI. Model 3: Adjusted for Model 2 variables plus smoking, alcohol, high-risk surgery, history of congestive heart failure, cerebrovascular disease, chronic pulmonary disease, any diabetes, and renal disease. OR, odds ratio; c.i., confidence interval; VTE, venous thromboembolism; CCI, Charlson Comorbidity Index.

When stratified by exercise frequency, individuals exercising 1–2 times per week showed numerically lower odds of VTE; however, this did not reach statistical significance in Model 1 (OR: 0.77, 95% c.i.: 0.47–1.23; *P* = 0.288), Model 2 (OR: 0.78, 95% c.i.: 0.45–1.25; *P* = 0.318), or Model 3 (OR: 0.79, 95% c.i.: 0.48–1.26; *P* = 0.334) (*Table 2* and *Table S5*). In contrast, individuals exercising ≥3 times per week exhibited a significantly lower risk of VTE across all models: Model 1 (OR: 0.64, 95% c.i.: 0.44–0.94; *P* = 0.021), Model 2 (OR: 0.65, 95% c.i.: 0.45–0.96; *P* = 0.028), and Model 3 (OR: 0.66, 95% c.i.: 0.45–0.96; *P* = 0.031). Furthermore, a significant dose-response relationship was observed in all models, with *P* for trend values of 0.021, 0.029, and 0.032 (*Table 2*). In a finer four-level analysis of exercise frequency, the adjusted odds ratio for VTE in Model 3 was 0.79 (95% c.i. 0.48–1.27; *P* = 0.340) for 1–2 sessions/week, 0.79 (95% c.i. 0.48–1.26; *P* = 0.325) for 3–4 sessions/week, and 0.58 (95% c.i. 0.36–0.90; *P* = 0.018) for ≥5 sessions/week, compared with no exercise, with a significant trend across categories (*P* for trend = 0.020; *Table S6*).

### Secondary outcomes: other postoperative outcomes and mortality

The analysis of secondary outcomes revealed that exercise status was significantly associated with a lower risk of certain postoperative complications, particularly DVT (*Table 3*). In the fully adjusted Model 3, being an exerciser was independently linked to a reduced risk of DVT compared to being a non-exerciser (OR: 0.66, 95% c.i.: 0.46–0.95; *P* = 0.025). When evaluating exercise frequency, individuals exercising ≥3 times per week demonstrated a significantly lower risk of DVT than those who did not exercise (OR: 0.61, 95% c.i.: 0.45–0.91; *P* = 0.016), with a significant dose-response relationship observed (*P* for trend = 0.016).

**Table 3:** Risk of postoperative outcomes according to exercise status and frequency (multivariable model 3)

Outcomes	Variable	Level	*Adjusted OR or HR (95% c.i.)	*P*-value	*P* for trend
DVT	Exercise status	Non-exerciser	1.00 (reference)		
		Exerciser	0.66 (0.46–0.95)	0.025	
	Exercise frequency	None	1.00 (reference)		0.016
		1–2/week	0.78 (0.47–1.27)	0.331	
		≥3/week	0.61 (0.45–0.91)	0.016	
Pneumonia	Exercise status	Non-exerciser	1.00 (reference)		
		Exerciser	1.04 (0.85–1.28)	0.729	
	Exercise frequency	None	1.00 (reference)		0.511
		1–2/week	0.96 (0.72–1.27)	0.779	
		≥3/week	1.07 (0.86–1.33)	0.558	
Surgical site infection	Exercise status	Non-exerciser	1.00 (reference)		
		Exerciser	0.92 (0.80–1.06)	0.239	
	Exercise frequency	None	1.00 (reference)		0.051
		1–2/week	1.03 (0.87–1.22)	0.736	
		≥3/week	0.87 (0.75–1.01)	0.073	
Stroke	Exercise status	Non-exerciser	1.00 (reference)		
		Exerciser	0.91 (0.81–1.02)	0.088	
	Exercise frequency	None	1.00 (reference)		0.299
		1–2/week	0.84 (0.72–0.99)	0.033	
		≥3/week	0.93 (0.83–1.05)	0.240	
1-year mortality	Exercise status	Non-exerciser	1.00 (reference)		
		Exerciser	0.92 (0.72–1.18)	0.518	
	Exercise frequency	None	1.00 (reference)		0.341
		1–2/week	1.03 (0.74–1.44)	0.861	
		≥3/week	0.89 (0.68–1.15)	0.365	
Overall mortality	Exercise status	Non-exerciser	1.00 (reference)		
		Exerciser	1.00 (0.95–1.06)	0.961	
	Exercise frequency	None	1.00 (reference)		0.680
		1–2/week	0.97 (0.90–1.06)	0.518	
		≥3/week	1.01 (0.95–1.07)	0.708	

DVT, deep vein thrombosis; OR, odds ratio; HR, hazard ratio; c.i., confidence interval. *Adjusted for age, sex, BMI, hemoglobin, systolic blood pressure, smoking, alcohol, high-risk surgery, history of congestive heart failure, cerebrovascular disease, chronic pulmonary disease, any diabetes, and renal disease.

**Figure 2:**
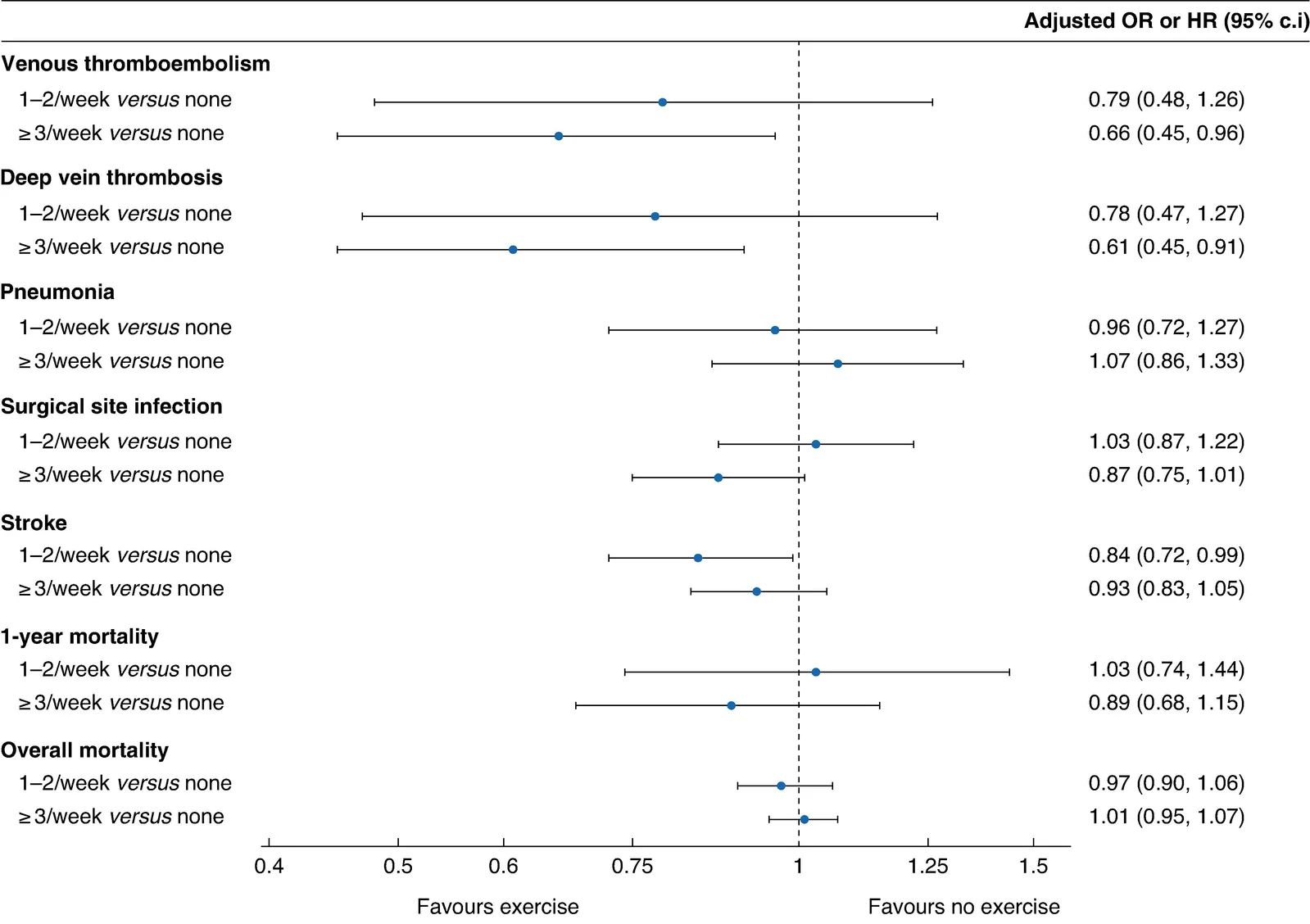
Forest plots depicting the adjusted associations between exercise frequency (none, 1–2, and ≥3 times/week) and postoperative outcomes

Regarding stroke, although overall exercise status did not reach statistical significance, a lower risk of stroke was specifically associated with exercising 1–2 times per week (OR: 0.84, 95% c.i.: 0.72–0.99; *P* = 0.033). Other postoperative outcomes, including pneumonia, surgical site infection, one-year mortality, and overall mortality, showed no significant associations with exercise status or frequency. These adjusted associations across clinical strata are visually summarized in the forest plots in *Fig. 2*.

### Subgroup analysis

Subgroup analyses stratified by age, sex, and lifestyle factors were conducted to assess the consistency of the association between exercise status and postoperative outcomes and to identify potential effect modifications (*Table 4* and *Table S7*). For the primary outcomes of VTE and DVT, the protective association with exercise was particularly pronounced in specific subgroups, with significant interactions observed for sex (P_int_ = 0.019 for VTE, P_int_ = 0.023 for DVT) and smoking status (P_int_ = 0.026 for VTE). Specifically, being an exerciser was independently associated with a lower risk of VTE (OR: 0.48, 95% c.i.: 0.30–0.77; *P* = 0.003) and DVT (OR: 0.46, 95% c.i.: 0.29–0.75; *P* = 0.002) among male patients, as well as among current or former smokers (OR: 0.40, 95% c.i.: 0.22–0.74; *P* = 0.003 for VTE, OR: 0.42, 95% c.i.: 0.23–0.77; *P* = 0.005 for DVT). While exercise was also associated with a lower risk of VTE (OR: 0.58, 95% c.i.: 0.36–0.93; *P* = 0.021) and DVT (OR: 0.53, 95% c.i.: 0.33–0.86; *P* = 0.009) in individuals younger than 65 years, the interaction *P*-values for age did not reach statistical significance (P_int_ = 0.209 and P_int_ = 0.147, respectively).

**Table 4:** Subgroup analysis of the association between exercise status and postoperative outcomes stratified by age, sex, and lifestyle factors

Outcomes	Subgroup Variables	Categories	Exercise status	*Adjusted OR or HR (95% c.i.)	*P* value	P_int_
VTE	Age	< 65 years	Non-exerciser	1.00 (reference)		0.209
			Exerciser	0.58 (0.36–0.93)	0.021	
		≥ 65 years	Non-exerciser	1.00 (reference)		
			Exerciser	0.83 (0.49–1.43)	0.487	
VTE	Sex	Female	Non-exerciser	1.00 (reference)		0.019
			Exerciser	1.07 (0.63–1.87)	0.819	
		Male	Non-exerciser	1.00 (reference)		
			Exerciser	0.48 (0.30–0.77)	0.003	
VTE	Smoking status	Non-smoker	Non-exerciser	1.00 (reference)		0.026
			Exerciser	0.92 (0.60–1.45)	0.715	
		Smoker	Non-exerciser	1.00 (reference)		
			Exerciser	0.40 (0.22–0.74)	0.003	
VTE	Frequency of drinking alcohol	Non-drinker	Non-exerciser	1.00 (reference)		0.412
			Exerciser	0.76 (0.49–1.20)	0.232	
		Drinker	Non-exerciser	1.00 (reference)		
			Exerciser	0.59 (0.34–1.06)	0.069	
DVT	Age	< 65 years	Non-exerciser	1.00 (reference)		0.147
			Exerciser	0.53 (0.33–0.86)	0.009	
		≥ 65 years	Non-exerciser	1.00 (reference)		
			Exerciser	0.83 (0.48–1.49)	0.530	
DVT	Sex	Female	Non-exerciser	1.00 (reference)		0.023
			Exerciser	1.03 (0.58–1.86)	0.933	
		Male	Non-exerciser	1.00 (reference)		
			Exerciser	0.46 (0.29–0.75)	0.002	
DVT	Smoking status	Non-smoker	Non-exerciser	1.00 (reference)		0.062
			Exerciser	0.85 (0.54–1.37)	0.497	
		Smoker	Non-exerciser	1.00 (reference)		
			Exerciser	0.42 (0.23–0.77)	0.005	
DVT	Frequency of drinking alcohol	Non-drinker	Non-exerciser	1.00 (reference)		0.491
			Exerciser	0.72 (0.45–1.15)	0.161	
		Drinker	Non-exerciser	1.00 (reference)		
			Exerciser	0.58 (0.33–1.05)	0.066	

OR, odds ratio; HR, hazard ratio; c.i., confidence interval. *Adjusted for age, sex, BMI, haemoglobin, systolic blood pressure, smoking, alcohol, high-risk surgery, history of congestive heart failure, cerebrovascular disease, chronic pulmonary disease, any diabetes, and renal disease.

Regarding the secondary outcomes detailed (*Table S7*), exercise was associated with a reduced risk of stroke specifically among patients aged 65 years or older (OR: 0.85, 95% c.i.: 0.73–0.99; *P* = 0.033) and non-smokers (OR: 0.87, 95% c.i.: 0.76–1.00; *P* = 0.044), although no significant interactions were detected for age (P_int_ = 0.537) or smoking status (P_int_ = 0.342). Furthermore, no significant associations or interactions were observed between exercise status and other outcomes, including surgical site infection, 1-year mortality, and overall mortality, across any analyzed subgroups.

## Discussion

This nationwide, population-based cohort study identified a significant and independent association between preoperative exercise and postoperative clinical outcomes, particularly for VTE and DVT. Patients who engaged in regular exercise prior to surgery exhibited a consistently lower risk of VTE and DVT compared to those in the non-exerciser group. A prominent finding of this study is the dose-response relationship between exercise frequency and thrombotic risk; individuals exercising at least three times per week demonstrated the most substantial risk reduction across all adjusted models. Although the associations with mortality and pneumonia did not reach statistical significance, a lower risk of stroke was observed among specific subgroups, including those exercising 1–2 times per week and elderly patients. These findings underscore the importance of assessing preoperative lifestyle factors during perioperative risk stratification, suggesting that exercise is a critical, modifiable determinant in preventing postoperative complications such as VTE.

The protective role of physical activity in cardiovascular health is well-documented in the general population; however, its specific impact during the perioperative period remains less understood. Existing literature has often treated physical activity as a secondary covariate or focused on small, single-centre cohorts undergoing specific procedures, limiting the generalizability of previous findings^18^. The present study addresses this gap by utilizing a nationally representative cohort of over 118 000 patients, enabling robust adjustments for demographic, institutional, and socioeconomic factors, as well as a broad range of comorbidities. The consistency of the findings—specifically, the independent association between exercise and lower VTE risk—suggests that preoperative exercise may be a meaningful predictor of postoperative recovery. Although randomized controlled trials are considered the gold standard, conducting an adequately powered randomized controlled trial to assess the impact of preoperative exercise on postoperative VTE would require a prohibitively large sample size due to the relatively low incidence of these events in non-cardiac surgery. This practical limitation highlights the critical and complementary role of large-scale observational analyses. By utilizing a nationwide, real-world database of over 118 000 patients, the current study provides robust evidence that would be logistically difficult to obtain through traditional clinical trials.

Several biological mechanisms likely underlie the observed association between exercise and a reduced risk of VTE and DVT. According to the classical Virchow’s triad, the development of venous thrombosis is driven by stasis, endothelial injury, and hypercoagulability^7^. Regular physical activity can directly mitigate these risks by enhancing the skeletal muscle pump mechanism, which improves venous return and reduces venous stasis^19,20^. Furthermore, chronic exercise has been shown to enhance fibrinolytic activity by increasing tissue-type plasminogen activator levels and decreasing plasminogen activator inhibitor-1 levels, thereby maintaining a more favourable antithrombotic profile^21,22^. The current data also showed that individuals who exercise generally have a lower burden of metabolic comorbidities, such as reduced systolic blood pressure and fasting glucose levels. However, the persistence of the protective effect after adjusting for these variables indicates that exercise provides direct haemodynamic and vascular benefits that extend beyond simple metabolic management.

A key finding of the current analysis is the frequency-dependent nature of the protective effect of exercise. A significant dose-response relationship was observed, with the odds of VTE progressively decreasing as exercise frequency increased. This dose–response relationship pertains specifically to exercise frequency; because intensity, duration, and type were not captured, the findings should not be interpreted as a gradient across total exercise volume. Notably, while exercising 1–2 times per week showed a numerical trend toward reduced risk, consistent statistical significance was primarily observed in individuals exercising three or more times per week. This suggests a possible threshold effect, whereby higher exercise frequency was associated with greater risk reduction; however, because intensity, duration, and type were not measured, a precise minimum volume of activity required to counteract the pro-thrombotic stress of surgery cannot be defined from these data^17,23,24^. Conversely, for stroke prevention, even lower exercise frequencies (1–2 times per week) were associated with reduced risk, particularly among elderly patients. This discrepancy likely reflects the differing pathophysiological mechanisms underlying stroke and VTE; whereas VTE is strongly influenced by stasis and coagulation, stroke risk is more closely linked to endothelial function and arterial stiffness, which may respond to even moderate increases in physical activity^23^.

The subgroup analyses revealed significant effect modifications by sex, smoking status, and age. The association between exercise and reduced risk of VTE/DVT was notably stronger in male patients and smokers, with significant interaction *P*-values observed in these groups. The greater benefit observed in males may be attributed to the higher baseline prevalence of high-risk surgeries in this population, providing more opportunity for the protective effects of exercise to manifest. In smokers, who inherently exist in a pro-thrombotic and pro-inflammatory state, exercise may partially counteract smoking-induced endothelial dysfunction, resulting in a more pronounced relative risk reduction compared to non-smokers^24,25^. Furthermore, exercise was associated with a lower risk of stroke, specifically in patients aged 65 years and older. Since aging is associated with increased arterial stiffening and reduced vascular compliance, physical activity may play a more critical role in maintaining cerebral perfusion and vascular health in older populations^26,27^.

The clinical profiles of the current cohort, particularly haemodynamic parameters such as systolic and diastolic blood pressure, provide important context for the baseline health of the study population. In the current analysis, blood pressure was included as a continuous covariate in the multivariable models, acknowledging its well-established role as a major risk factor for cardiovascular events. Generally, higher blood pressure is associated with increased arterial stiffness, whereas lower blood pressure levels in surgical patients may indicate frailty or underlying chronic illness—factors often inversely correlated with a patient’s capacity to exercise^28,29^. By adjusting for these baseline haemodynamic parameters, it was confirmed that the inverse association between preoperative exercise and VTE risk remained independent of initial blood pressure levels.

It should be emphasised that the exposure was habitual preoperative exercise captured as a baseline characteristic, not a prehabilitation intervention; accordingly, these findings identify exercise frequency as a modifiable risk marker and provide a rationale for interventional prehabilitation studies, rather than directly demonstrating the efficacy of a prehabilitation programme. With this caveat, the clinical implications of this study support the rationale for evaluating preoperative prehabilitation programs^8,9^. Surgeons and clinicians may consider preoperative physical activity not only as a marker of baseline health but also as a target for intervention. Encouraging patients to engage in exercise at least three times per week in the weeks or months leading up to an elective procedure may enhance physiological reserve, and whether this translates into a lower risk of thrombotic complications warrants prospective evaluation. Given the consistency of the current findings across various surgical types and patient groups, exercise status should be incorporated into standard preoperative assessments. Moreover, because the exposure reflects self-reported exercise frequency over an unspecified period before predominantly elective surgery, these findings should not be translated directly into specific prehabilitation exercise prescriptions; rather, they support the rationale for prospective interventional studies.

Several limitations must be acknowledged. First, exercise frequency was based on self-reported data and may be subject to recall bias, although exercise frequency is generally recalled more reliably than exercise intensity or duration. Moreover, Korean National Health Insurance claims data contain no billing or procedure codes for structured prehabilitation programmes or prescribed exercise, as these are not separately reimbursed; patients who may have undergone formal prehabilitation could therefore not be identified or analysed separately, and the exposure reflects habitual baseline exercise rather than a prescribed peri-referral intervention. Second, the observational nature of the claims database means that residual confounding from unmeasured variables—such as surgical complexity, intraoperative physiological parameters, or specific anaesthesia types—cannot be entirely ruled out. In particular, formal VTE risk-assessment scores such as the Caprini score or the UK Department of Health tool are clinical bedside instruments that are not captured in administrative claims data and could not be incorporated; however, the multivariable models adjusted for the principal constituents of these scores, including age, sex, malignancy, overall Charlson comorbidity burden, surgery type, high-risk surgery status, and major comorbidities, thereby capturing much of the construct these tools represent, although not their exact scoring. In addition, mechanical and pharmacological thromboprophylaxis could not be directly ascertained: mechanical prophylaxis has no reliable, separately billable code in Korean claims data, and although pharmacological prophylaxis is in principle identifiable from prescription records, perioperative prophylaxis in Korea is largely protocol-driven according to surgery type and risk category and was therefore not modelled as a separate covariate. Third, the study population was drawn from a single nationwide database with a relatively homogeneous demographic, potentially limiting the generalizability of the findings to other healthcare systems or ethnic groups. Fourth, the screening questionnaire captured exercise frequency only; the absence of data on exercise intensity, duration, and type—the remaining components of the FITT (frequency, intensity, time, type) framework—precludes analysis of total physical activity volume, and any observed dose–response relationship therefore pertains to frequency alone. Fifth, the NHIS-NSC sample-cohort data do not reliably distinguish surgical urgency, and elective *versus* emergency status could therefore not be modelled separately. Finally, given the observational design, all findings represent associations and should not be interpreted as demonstrating causation.

This nationwide study demonstrates that preoperative exercise is associated with a significantly lower risk of postoperative VTE and DVT, exhibiting a clear dose-response relationship. Engaging in three or more exercise sessions per week appears to offer the most substantial protection. These findings highlight the critical importance of physical activity as an independent predictor of surgical outcomes and provide a strong rationale for implementing preoperative interventions aimed at increasing physical activity to enhance postoperative recovery and patient safety.

## Supplementary Material

zrag109_Supplementary_Data

## Data Availability

The data used in this study are available from the National Health Insurance Service (NHIS) of South Korea and can be accessed by qualified researchers upon reasonable request and approval through the NHIS data access process. These data are not publicly available due to data protection regulations.
