# Supplementary material for: Association between preoperative physical exercise and the risk of postoperative venous thromboembolism: nationwide population-based cohort study
Source: BJS Open. 2026 Sep 10;10(5):zrag109. doi: 10.1093/bjsopen/zrag109 (PMC13560078; doi:10.1093/bjsopen/zrag109)
Supplement: Supplementary file 1 [file zrag109_supplementary_data.docx]

**Association between preoperative physical exercise and the risk of postoperative venous thromboembolism: nationwide population-based cohort study**

**Chan-Sik Kim, MD, PhD^1,^ *, Bumwoo Park, PhD^2^, Hyeong-Seok Yoon, MD^3^, Ji-Hoon Sim, MD, PhD^1,^ ***

^1^Department of Anesthesiology and Pain Medicine, Asan Medical Center, University of Ulsan College of Medicine, Seoul, Republic of Korea

^2^Department of Information Medicine, Asan Medical Center, University of Ulsan College of Medicine, Seoul, Republic of Korea

^3^Department of Anesthesiology and Pain Medicine, Gangneung Asan Hospital, University of Ulsan College of Medicine, Gangneung, Republic of Korea

**Correspondence:**

Ji-Hoon Sim, MD, PhD. Email: [jihoon_sim@amc.seoul.kr](mailto:jihoon_sim@amc.seoul.kr)

**Supplementary Materials - Index**

| **Supplementary Tables** |  |
| --- | --- |
| Supplementary Table 1 | *page 2* |
| Supplementary Table 2  Supplementary Table 3  Supplementary Table 4  Supplementary Table 5  Supplementary Table 6  Supplementary Table 7 | *page 4*  *page 5*  *page 7*  *page 8*  *page 9*  *page 10* |
|  |  |

**Supplementary Table 1.** ICD-10 codes used to define comorbidities for calculating the Charlson Comorbidity Index

| **Comorbidities** | **ICD-10 code** |
| --- | --- |
| Myocardial infarction | I21.x, I22.x, I25.2 |
| Congestive heart failure | I09.9, I11.0, I13.0, I13.2, I25.5, I42.0, I42.5–I42.9, I43.x, I50.x, P29.0 |
| Peripheral vascular disease | I70.x, I71.x, I73.1, I73.8, I73.9, I77.1, I79.0, I79.2, K55.1, K55.8, K55.9, Z95.8, Z95.9 |
| Cerebrovascular disease | G45.x, G46.x, H34.0, I60.x–I69.x |
| Dementia | F00.x–F03.x, F05.1, G30.x, G31.1 |
| Chronic pulmonary disease | I27.8, I27.9, J40.x–J47.x, J60.x–J67.x, J68.4, J70.1, J70.3 |
| Rheumatic disease | M05.x, M06.x, M31.5, M32.x–M34.x, M35.1, M35.3, M36.0 |
| Peptic ulcer disease | K25.x–K28.x |
| Mild liver disease | B18.x, K70.0–K70.3, K70.9, K71.3–K71.5, K71.7, K73.x, K74.x, K76.0, K76.2–K76.4, K76.8, K76.9, Z94.4 |
| Diabetes without chronic complications | E10.0, E10.1, E10.6, E10.8, E10.9, E11.0, E11.1, E11.6, E11.8, E11.9, E12.0, E12.1, E12.6, E12.8, E12.9, E13.0, E13.1, E13.6, E13.8, E13.9, E14.0, E14.1, E14.6, E14.8, E14.9 |
| Diabetes with chronic complications | E10.2–E10.5, E10.7, E11.2–E11.5, E11.7, E12.2–E12.5, E12.7, E13.2–E13.5, E13.7, E14.2–E14.5, E14.7 |
| Hemiplegia or paraplegia | G04.1, G11.4, G80.1, G80.2, G81.x, G82.x, G83.0–G83.4, G83.9 |
| Renal disease | I12.0, I13.1, N03.2–N03.7, N05.2–N05.7, N18.x, N19.x, N25.0, Z49.0–Z49.2, Z94.0, Z99.2 |
| Any malignancy (excluding non-melanoma skin cancers) | C00.x–C26.x, C30.x–C34.x, C37.x–C41.x, C43.x, C45.x–C58.x, C60.x–C76.x, C81.x–C85.x, C88.x, C90.x–C97.x |
| Moderate or severe liver disease | I85.0, I85.9, I86.4, I98.2, K70.4, K71.1, K72.1, K72.9, K76.5, K76.6, K76.7 |
| Metastatic solid tumor | C77.x–C80.x |
| AIDS/HIV | B20.x–B22.x, B24× |

AIDS, acquired immunodeficiency syndrome; HIV, human immunodeficiency virus.

**Description**

This table lists the International Classification of Diseases, Tenth Revision (ICD-10) codes used to identify individual comorbid conditions for calculating the Charlson Comorbidity Index in this study. Comorbidities were determined from diagnosis codes recorded prior to surgery and classified according to established claims-based definitions.

**Supplementary Table 2.** Definitions and ICD-10 codes used for postoperative outcomes

| **Postoperative outcomes** | **Definitions and ICD-10 code** |
| --- | --- |
| Venous thromboembolism | A composite outcome of pulmonary embolism (I26.x) and deep vein thrombosis (I80.x) |
| Deep vein thrombosis | I80.x |
| Stroke | I60.x–I62.x (Hemorrhagic), I63.x (Ischemic), I64.x (Unspecified) |
| Pneumonia | J12.x–J18.x, J69.0 |
| Surgical site infection | T81.4, O86.0 |
| 30-day readmission | Any inpatient admission occurring within 30 days after discharge from the index operation stay; determined based on admission and discharge dates |
| 90-day mortality | All-cause death within 90 days after the index operation date; derived from the death registry field |
| 1-year mortality | All-cause death within 1 year after index operation date; derived from death registry field |
| Overall mortality | All-cause death within the entire follow-up period after index operation date; derived from death registry field |

**Description**

This table lists the International Classification of Diseases, Tenth Revision (ICD-10) codes and operational definitions used to identify postoperative complications occurring within 30 days after the index operation. Comorbidities were determined based on diagnosis codes recorded within 30 days post-surgery in patients who had no corresponding diagnoses during the preoperative lookback period.

**Supplementary Table 3.** Baseline characteristics according to exercise frequency (3-level: none, 1–2, and ≥3 times/week)

|  | **Total study population** | | | | | |
| --- | --- | --- | --- | --- | --- | --- |
|  | **Exercise frequency** | | | | | |
|  | **Total  (n = 118,340)** | **None  (n = 33,351)** | **1–2 times/week  (n = 27,275)** | **≥3 times/week  (n = 57,714)** | **P value** | **SMD** |
| **Demographic variables** |  |  |  |  |  |  |
| Age, years | 50.0 ± 14.0 | 52.3 ± 13.7 | 47.2 ± 13.0 | 50.0 ± 14.4 | <0.001 | 0.382 |
| Male sex | 65,872 (55.7%) | 17,416 (52.2%) | 15,961 (58.5%) | 32,495 (56.3%) | <0.001 | 0.127 |
| BMI, kg/m^2^ | 23.9 ± 3.3 | 23.9 ± 3.3 | 23.9 ± 3.3 | 23.9 ± 3.2 | 0.119 | 0.015 |
| Waist circumference, cm | 80.9 ± 9.2 | 81.1 ± 9.2 | 80.8 ± 9.3 | 80.8 ± 9.1 | <0.001 | 0.035 |
| Systolic blood pressure, mmHg | 122.6 ± 14.8 | 123.2 ± 15.1 | 121.8 ± 14.5 | 122.7 ± 14.8 | <0.001 | 0.095 |
| Diastolic blood pressure, mmHg | 76.4 ± 10.0 | 76.6 ± 10.0 | 76.2 ± 10.0 | 76.3 ± 9.9 | <0.001 | 0.036 |
| Hemoglobin | 14.0 ± 1.6 | 13.9 ± 1.6 | 14.2 ± 1.6 | 14.0 ± 1.6 | <0.001 | 0.152 |
| Fasting glucose | 99.1 ± 24.9 | 99.7 ± 25.4 | 98.3 ± 24.3 | 99.1 ± 25.0 | <0.001 | 0.058 |
| Total cholesterol | 194.9 ± 40.0 | 195.9 ± 40.1 | 195.5 ± 42.0 | 194.0 ± 39.1 | <0.001 | 0.047 |
| AST | 25.7 ± 25.5 | 26.0 ± 22.5 | 25.4 ± 16.5 | 25.7 ± 30.3 | 0.014 | 0.030 |
| ALT | 25.6 ± 38.8 | 25.7 ± 28.2 | 26.3 ± 25.9 | 25.2 ± 48.1 | <0.001 | 0.029 |
| GGT | 38.1 ± 52.4 | 38.8 ± 55.5 | 38.9 ± 49.4 | 37.2 ± 51.8 | <0.001 | 0.033 |
| Institutional case volume |  |  |  |  | 0.128 | 0.015 |
| <100 beds | 114,128 (96.4%) | 32,190 (96.5%) | 26,261 (96.3%) | 55,677 (96.5) |  |  |
| 100–499 beds | 1786 (1.5) | 519 (1.6) | 434 (1.6) | 833 (1.4) |  |  |
| ≥500 beds | 2426 (2.1) | 642 (1.9) | 580 (2.1) | 1204 (2.1) |  |  |
| Insurance type |  |  |  |  | <0.001 | 0.122 |
| Regional insured | 26,451 (22.4) | 8293 (24.9) | 5394 (19.8) | 12,764 (22.1) |  |  |
| Workplace insured | 91,889 (77.6) | 25,058 (75.1) | 21,881 (80.2) | 44,950 (77.9) |  |  |
| Income level |  |  |  |  | <0.001 | 0.114 |
| Q1 | 17,391 (14.7) | 5383 (16.1) | 3582 (13.1) | 8426 (14.6) |  |  |
| Q2 | 24,392 (20.6) | 7041 (21.1) | 5271 (19.3) | 12,080 (20.9) |  |  |
| Q3 | 39,420 (33.3) | 11,053 (33.1) | 9277 (34.0) | 19,090 (33.1) |  |  |
| Q4 | 37,137 (31.4) | 9874 (29.6) | 9145 (33.5) | 18,118 (31.4) |  |  |
| Year of surgery |  |  |  |  | <0.001 | 0.147 |
| 2009–2010 | 33,846 (28.6) | 10,583 (31.7) | 7803 (28.6) | 15,460 (26.8) |  |  |
| 2011–2012 | 36,706 (31.0) | 10,668 (32.0) | 8374 (30.7) | 17,664 (30.6) |  |  |
| 2013–2014 | 28,634 (24.2) | 7539 (22.6) | 6704 (24.6) | 14,391 (24.9) |  |  |
| 2015–2016 | 19,154 (16.2) | 4561 (13.7) | 4394 (16.1) | 10,199 (17.7) |  |  |
| Smoking status |  |  |  |  | <0.001 | 0.146 |
| Non-smoker | 68428 (57.8) | 20515 (61.5) | 14911 (54.7) | 33002 (57.2) |  |  |
| Ex-smoker | 19389 (16.4) | 4448 (13.3) | 4703 (17.2) | 10238 (17.7) |  |  |
| Current smoker | 30523 (25.8) | 8388 (25.2) | 7661 (28.1) | 14474 (25.1) |  |  |
| Drinking frequency |  |  |  |  | <0.001 | 0.362 |
| None | 60095 (50.8) | 19648 (58.9) | 12031 (44.1) | 28416 (49.2) |  |  |
| 1–2 times/week | 40769 (34.5) | 8797 (26.4) | 11542 (42.3) | 20430 (35.4) |  |  |
| 3–4 times/week | 12491 (10.6) | 3196 (9.6) | 2852 (10.5) | 6443 (11.2) |  |  |
| ≥5 times/week | 4985 (4.2) | 1710 (5.1) | 850 (3.1) | 2425 (4.2) |  |  |
| Type of surgery |  |  |  |  | <0.001 | 0.102 |
| General | 14809 (12.5) | 4350 (13.0) | 3304 (12.1) | 7155 (12.4) |  |  |
| Otolaryngology | 24553 (20.7) | 6349 (19.0) | 6065 (22.2) | 12139 (21.0) |  |  |
| Urologic | 10320 (8.7) | 2840 (8.5) | 2558 (9.4) | 4922 (8.5) |  |  |
| Orthopedic | 42739 (36.1) | 12555 (37.6) | 9463 (34.7) | 20721 (35.9) |  |  |
| Gynecologic | 11343 (9.6) | 3295 (9.9) | 2611 (9.6) | 5437 (9.4) |  |  |
| Neurosurgery | 4936 (4.2) | 1486 (4.5) | 1083 (4.0) | 2367 (4.1) |  |  |
| Cardiothoracic | 1007 (0.9) | 284 (0.9) | 212 (0.8) | 511 (0.9) |  |  |
| Other surgeries | 8633 (7.3) | 2192 (6.6) | 1979 (7.3) | 4462 (7.7) |  |  |
| High-risk surgery (general, orthopedic, neurosurgery, and cardiothoracic surgery) | 63491 (53.7) | 18675 (56.0) | 14062 (51.6) | 30754 (53.3) | <0.001 | 0.089 |
| CCI score |  |  |  |  | <0.001 | 0.344 |
| 0–2 | 103,499 (87.5) | 65,402 (89.6) | 3,055 (77.1) | 17,305 (88.4) |  |  |
| 3–4 | 11,281 (9.5) | 5,819 (8.0) | 642 (16.2) | 1,796 (9.2) |  |  |
| ≥5 | 3,560 (3.0) | 1,778 (2.4) | 266 (6.7) | 476 (2.4) |  |  |
| **Outcomes** |  |  |  |  |  |  |
| MACCE | 2173 (1.8) | 775 (2.3) | 344 (1.3) | 1054 (1.8) | <0.001 |  |
| Myocardial infarction | 206 (0.2) | 58 (0.2) | 29 (0.1) | 119 (0.2) | 0.005 |  |
| Stroke | 1978 (1.7) | 720 (2.2) | 317 (1.2) | 941 (1.6) | <0.001 |  |
| Pneumonia | 431 (0.4) | 133 (0.4) | 80 (0.3) | 218 (0.4) | 0.075 |  |
| Surgical site infection | 986 (0.8) | 306 (0.9) | 235 (0.9) | 445 (0.8) | 0.054 |  |
| Urinary tract infection | 1220 (1.0) | 383 (1.1) | 239 (0.9) | 598 (1.0) | 0.004 |  |
| VTE | 133 (0.1) | 54 (0.2) | 25 (0.1) | 54 (0.1) | 0.006 |  |
| DVT | 123 (0.1) | 51 (0.2) | 24 (0.1) | 48 (0.1) | 0.005 |  |
| 30-day readmission | 827 (0.7) | 239 (0.7) | 199 (0.7) | 389 (0.7) | 0.596 |  |
| 90-day mortality | 56 (0.0) | 13 (0.0) | 12 (0.0) | 31 (0.1) | 0.591 |  |
| 1-year mortality | 288 (0.2) | 102 (0.3) | 53 (0.2) | 133 (0.2) | 0.015 |  |
| Overall mortality | 5381 (4.5) | 2001 (6.0) | 866 (3.2) | 2514 (4.4) | <0.001 |  |

Values are expressed as mean ± standard deviation or n (proportion).

SMD, standardized mean difference; BMI, body mass index; AST, Aspartate aminotransferase; ALT: Alanine aminotransferase; GGT: Gamma-glutamyl transferase; HIV, human immunodeficiency virus; AIDS, acquired immunodeficiency syndrome; CCI, Charlson comorbidity index; MACCE, major adverse cardiac and cerebrovascular events; DVT, deep vein thrombosis; VTE, venous thromboembolism.

**Supplementary Table 4.** Logistic regression analysis of risk factors for the VTE risk according to exercise status (Binary: non-exerciser vs. exerciser)

|  | **Multivariable model 1** | | **Multivariable mode 2** | | **Multivariable model 3** | |
| --- | --- | --- | --- | --- | --- | --- |
|  | **Adjusted OR (95% CI)** | **P value** | **Adjusted OR (95% CI)** | **P value** | **Adjusted OR (95% CI)** | **P value** |
| Exercise frequency |  |  |  |  |  |  |
| Non-exerciser | 1.00 (reference) |  | 1.00 (reference) |  | 1.00 (reference) |  |
| Exerciser | 0.68 (0.48–0.96) | 0.027 | 0.69 (0.49–0.98) | 0.037 | 0.69 (0.49–0.99) | 0.041 |
| Age | 1.06 (1.05–1.08) | <0.001 | 1.05 (1.03–1.06) | <0.001 | 1.04 (1.03–1.06) | <0.001 |
| Male sex | 1.15 (0.82–1.63) | 0.415 | 1.62 (1.08–2.44) | 0.020 | 1.69 (1.03–2.73) | 0.035 |
| BMI |  |  | 1.13 (1.08–1.19) | <0.001 | 1.12 (1.07–1.18) | <0.001 |
| Hemoglobin |  |  | 0.83 (0.73–0.95) | 0.004 | 0.84 (0.74–0.96) | 0.008 |
| Systolic blood pressure |  |  | 1.00 (0.99–1.02) | 0.493 | 1.00 (0.99–1.01) | 0.634 |
| CCI score |  |  |  |  |  |  |
| 0–2 |  |  | 1.00 (reference) |  |  |  |
| 3–4 |  |  | 2.07 (1.34–3.13) | <0.001 |  |  |
| ≥5 |  |  | 2.81 (1.59–4.73) | <0.001 |  |  |
| Smoking status |  |  |  |  |  |  |
| Non-smoker |  |  |  |  | 1.00 (reference) |  |
| Ex-smoker |  |  |  |  | 0.76 (0.44–1.29) | 0.314 |
| Current smoker |  |  |  |  | 0.72 (0.41–1.25) | 0.246 |
| Drinking frequency |  |  |  |  |  |  |
| None |  |  |  |  | 1.00 (reference) |  |
| 1–2 times/week |  |  |  |  | 1.20 (0.74–1.90) | 0.454 |
| 3–4 times/week |  |  |  |  | 1.43 (0.74–2.61) | 0.264 |
| ≥5 times/week |  |  |  |  | 1.27 (0.52–2.70) | 0.562 |
| High-risk surgery |  |  |  |  | 1.24 (0.87–1.79) | 0.244 |
| Congestive heart failure |  |  |  |  | 3.92 (2.31–6.40) | <0.001 |
| Cerebrovascular disease |  |  |  |  | 0.83 (0.43–1.45) | 0.529 |
| Chronic pulmonary disease |  |  |  |  | 1.36 (0.93–1.97) | 0.103 |
| Diabetes |  |  |  |  | 1.88 (1.28–2.75) | 0.001 |
| Renal disease |  |  |  |  | 1.57 (0.69–3.13) | 0.238 |

Model 1: Adjusted for age and sex. Model 2: Adjusted for Model 1 variables plus BMI, hemoglobin, systolic blood pressure, and CCI. Model 3: Adjusted for Model 2 variables plus smoking, alcohol, high-risk surgery, history of congestive heart failure, cerebrovascular disease, chronic pulmonary disease, any diabetes, and renal disease.

OR, odds ratio; CI, confidence interval; VTE, venous thromboembolism; CCI, Charlson Comorbidity Index. **Supplementary Table 5.** Logistic regression analysis for VTE risk according to exercise frequency (3-level: none, 1–2, and ≥3 times/week)

|  | **Multivariable model 1** | | | **Multivariable mode 2** | | | **Multivariable model 3** | | |
| --- | --- | --- | --- | --- | --- | --- | --- | --- | --- |
|  | **Adjusted OR (95% CI)** | **P value** | **Overall P value** | **Adjusted OR (95% CI)** | **P value** | **Overall P value** | **Adjusted OR (95% CI)** | **P value** | **Overall P value** |
| Exercise frequency |  |  | 0.071 |  |  | 0.091 |  |  | 0.100 |
| None | 1.00 (reference) |  |  | 1.00 (reference) |  |  | 1.00 (reference) |  |  |
| 1–2 times/week | 0.77 (0.47–1.23) | 0.288 |  | 0.78 (0.45–1.25) | 0.318 |  | 0.79 (0.48–1.26) | 0.334 |  |
| ≥3 times/week | 0.64 (0.44–0.94) | 0.021 |  | 0.65 (0.45–0.96) | 0.028 |  | 0.66 (0.45–0.96) | 0.031 |  |
| Age | 1.06 (1.05–1.08) | <0.001 |  | 1.05 (1.03–1.06) | <0.001 |  | 1.04 (1.03–1.06) | <0.001 |  |
| Male sex | 1.16 (0.82–1.63) | 0.410 |  | 1.62 (1.08–2.44) | 0.020 |  | 1.69 (1.03–2.74) | 0.034 |  |
| BMI |  |  |  | 1.13 (1.08–1.19) | <0.001 |  | 1.12 (1.07–1.18) | <0.001 |  |
| Hemoglobin |  |  |  | 0.83 (0.73–0.95) | 0.004 |  | 0.84 (0.74–0.96) | 0.007 |  |
| Systolic blood pressure |  |  |  | 1.00 (0.99–1.02) | 0.493 |  | 1.00 (0.99–1.01) | 0.629 |  |
| CCI score |  |  |  |  |  | <0.001 |  |  |  |
| 0–2 |  |  |  | 1.00 (reference) |  |  |  |  |  |
| 3–4 |  |  |  | 2.07 (1.35–3.13) | <0.001 |  |  |  |  |
| ≥5 |  |  |  | 2.81 (1.59–4.72) | <0.001 |  |  |  |  |
| Smoking status |  |  |  |  |  |  |  |  | 0.096 |
| Non-smoker |  |  |  |  |  |  | 1.00 (reference) |  |  |
| Ex-smoker |  |  |  |  |  |  | 0.76 (0.44–1.29) | 0.314 |  |
| Current smoker |  |  |  |  |  |  | 0.72 (0.40–1.24) | 0.243 |  |
| Drinking frequency |  |  |  |  |  |  |  |  | 0.305 |
| None |  |  |  |  |  |  | 1.00 (reference) |  |  |
| 1–2 times/week |  |  |  |  |  |  | 1.19 (0.74–1.89) | 0.465 |  |
| 3–4 times/week |  |  |  |  |  |  | 1.43 (0.74–2.62) | 0.260 |  |
| ≥5 times/week |  |  |  |  |  |  | 1.28 (0.52–2.71) | 0.556 |  |
| High-risk surgery |  |  |  |  |  |  | 1.24 (0.87–1.79) | 0.244 |  |
| Congestive heart failure |  |  |  |  |  |  | 3.92 (2.30–6.39) | <0.001 |  |
| Cerebrovascular disease |  |  |  |  |  |  | 0.83 (0.43–1.45) | 0.528 |  |
| Chronic pulmonary disease |  |  |  |  |  |  | 1.36 (0.93–1.97) | 0.104 |  |
| Diabetes |  |  |  |  |  |  | 1.89 (1.28–2.75) | 0.001 |  |
| Renal disease |  |  |  |  |  |  | 1.57 (0.69–3.13) | 0.238 |  |

Model 1: Adjusted for age and sex. Model 2: Adjusted for variables in Model 1 plus BMI, hemoglobin, systolic blood pressure, and CCI. Model 3: Adjusted for variables in Model 2 plus smoking, alcohol, high-risk surgery, history of congestive heart failure, cerebrovascular disease, chronic pulmonary disease, any diabetes, and renal disease.

OR, odds ratio; CI, confidence interval; VTE, venous thromboembolism; CCI, Charlson Comorbidity Index.

**Supplementary Table 6. Logistic regression analysis for VTE risk according to exercise frequency (4-level: none, 1–2, 3–4, and ≥5 times/week)**

| **Exercise frequency** | **Adjusted OR (95% CI)** | **P value** |
| --- | --- | --- |
| None | 1.00 (reference) |  |
| 1–2 times/week | 0.79 (0.48–1.27) | 0.340 |
| 3–4 times/week | 0.79 (0.48–1.26) | 0.325 |
| ≥5 times/week | 0.58 (0.36–0.90) | 0.018 |

Model 3: Adjusted for age, sex, BMI, hemoglobin, systolic blood pressure, CCI, smoking, alcohol, high-risk surgery, and history of congestive heart failure, cerebrovascular disease, chronic pulmonary disease, any diabetes, and renal disease. P for trend = 0.020.

OR, odds ratio; CI, confidence interval; VTE, venous thromboembolism; CCI, Charlson Comorbidity Index.

**Supplementary Table 7.** Subgroup analysis for the association between exercise status and other postoperative outcomes stratified by age, sex, and lifestyle factors

| **Outcomes** | **Subgroup Variables** | **Categories** | **Exercise status** | ***Adjusted OR or HR (95% CI)** | **P value** | **P_int_** |
| --- | --- | --- | --- | --- | --- | --- |
| Stroke | Age | < 65 years | Non-exerciser | 1.00 (reference) |  | 0.537 |
|  |  |  | Exerciser | 0.94 (0.79–1.12) | 0.488 |  |
|  |  | ≥ 65 years | Non-exerciser | 1.00 (reference) |  |  |
|  |  |  | Exerciser | 0.85 (0.73–0.99) | 0.033 |  |
| Stroke | Sex | Female | Non-exerciser | 1.00 (reference) |  | 0.654 |
|  |  |  | Exerciser | 0.93 (0.79–1.09) | 0.361 |  |
|  |  | Male | Non-exerciser | 1.00 (reference) |  |  |
|  |  |  | Exerciser | 0.89 (0.75–1.05) | 0.151 |  |
| Stroke | Smoking status | Non-smoker | Non-exerciser | 1.00 (reference) |  | 0.342 |
|  |  |  | Exerciser | 0.87 (0.76–1.00) | 0.044 |  |
|  |  | Smoker | Non-exerciser | 1.00 (reference) |  |  |
|  |  |  | Exerciser | 1.00 (0.82–1.23) | 0.998 |  |
| Stroke | Drinking frequency | Non-drinker | Non-exerciser | 1.00 (reference) |  | 0.863 |
|  |  |  | Exerciser | 0.89 (0.78–1.02) | 0.100 |  |
|  |  | Drinker | Non-exerciser | 1.00 (reference) |  |  |
|  |  |  | Exerciser | 0.94 (0.75–1.17) | 0.554 |  |
|  |  |  | Exerciser | 0.97 (0.90–1.04) | 0.355 |  |
|  |  | Drinker | Non-exerciser | 1.00 (reference) |  |  |
|  |  |  | Exerciser | 1.06 (0.96–1.17) | 0.241 |  |
| Surgical site infection | Age | <65 | Non-exerciser | 1.00 (reference) |  | 0.559 |
|  |  |  | Exerciser | 0.95 (0.81–1.12) | 0.529 |  |
|  |  | ≥65 | Non-exerciser | 1.00 (reference) |  |  |
|  |  |  | Exerciser | 0.83 (0.65–1.08) | 0.168 |  |
| Surgical site infection | Sex | Female | Non-exerciser | 1.00 (reference) |  | 0.254 |
|  |  |  | Exerciser | 0.99 (0.81–1.22) | 0.926 |  |
|  |  | Male | Non-exerciser | 1.00 (reference) |  |  |
|  |  |  | Exerciser | 0.86 (0.72–1.04) | 0.115 |  |
| Surgical site infection | Smoking status | Non-smoker | Non-exerciser | 1.00 (reference) |  | 0.917 |
|  |  |  | Exerciser | 0.93 (0.78–1.12) | 0.443 |  |
|  |  | Smoker | Non-exerciser | 1.00 (reference) |  |  |
|  |  |  | Exerciser | 0.90 (0.73–1.13) | 0.362 |  |
| Surgical site infection | Drinking frequency | Non-drinker | Non-exerciser | 1.00 (reference) |  | 0.843 |
|  |  |  | Exerciser | 0.91 (0.76–1.09) | 0.311 |  |
|  |  | Drinker | Non-exerciser | 1.00 (reference) |  |  |
|  |  |  | Exerciser | 0.93 (0.75–1.15) | 0.505 |  |
| 1-year mortality | Age | <65 | Non-exerciser | 1.00 (reference) |  | 0.868 |
|  |  |  | Exerciser | 0.91 (0.62–1.33) | 0.618 |  |
|  |  | ≥65 | Non-exerciser | 1.00 (reference) |  |  |
|  |  |  | Exerciser | 0.86 (0.63–1.18) | 0.357 |  |
| 1-year mortality | Sex | Female | Non-exerciser | 1.00 (reference) |  | 0.074 |
|  |  |  | Exerciser | 0.66 (0.41–1.07) | 0.094 |  |
|  |  | Male | Non-exerciser | 1.00 (reference) |  |  |
|  |  |  | Exerciser | 1.04 (0.78–1.38) | 0.799 |  |
| 1-year mortality | Smoking status | Non-smoker | Non-exerciser | 1.00 (reference) |  | 0.221 |
|  |  |  | Exerciser | 0.79 (0.56–1.11) | 0.182 |  |
|  |  | Smoker | Non-exerciser | 1.00 (reference) |  |  |
|  |  |  | Exerciser | 1.03 (0.72–1.46) | 0.878 |  |
| 1-year mortality | Drinking frequency | Non-drinker | Non-exerciser | 1.00 (reference) |  | 0.175 |
|  |  |  | Exerciser | 0.82 (0.61–1.10) | 0.195 |  |
|  |  | Drinker | Non-exerciser | 1.00 (reference) |  |  |
|  |  |  | Exerciser | 1.16 (0.74–1.83) | 0.510 |  |
| Overall mortality | Age | <65 | Non-exerciser | 1.00 (reference) |  | 0.266 |
|  |  |  | Exerciser | 1.05 (0.95–1.16) | 0.348 |  |
|  |  | ≥65 | Non-exerciser | 1.00 (reference) |  |  |
|  |  |  | Exerciser | 0.98 (0.91–1.05) | 0.533 |  |
| Overall mortality | Sex | Female | Non-exerciser | 1.00 (reference) |  | 0.172 |
|  |  |  | Exerciser | 0.96 (0.87–1.05) | 0.354 |  |
|  |  | Male | Non-exerciser | 1.00 (reference) |  |  |
|  |  |  | Exerciser | 1.03 (0.96–1.10) | 0.412 |  |
| Overall mortality | Smoking status | Non-smoker | Non-exerciser | 1.00 (reference) |  | 0.810 |
|  |  |  | Exerciser | 1.00 (0.92–1.08) | 0.933 |  |
|  |  | Smoker | Non-exerciser | 1.00 (reference) |  |  |
|  |  |  | Exerciser | 1.01 (0.93–1.09) | 0.843 |  |
| Overall mortality | Drinking frequency | Non-drinker | Non-exerciser | 1.00 (reference) |  | 0.071 |
|  |  |  | Exerciser | 0.97 (0.90–1.04) | 0.355 |  |
|  |  | Drinker | Non-exerciser | 1.00 (reference) |  |  |
|  |  |  | Exerciser | 1.06 (0.96–1.17) | 0.241 |  |

* Adjusted for age, sex, BMI, hemoglobin, systolic blood pressure, smoking, alcohol, high-risk surgery, history of congestive heart failure, cerebrovascular disease, chronic pulmonary disease, any diabetes, and renal disease.

OR, odds ratio; HR, hazard ratio; CI, confidence interval.
